# Micronutrients supplementation and nutritional status in cognitively impaired elderly persons: a two-month open label pilot study

**DOI:** 10.1186/1475-2891-12-148

**Published:** 2013-11-15

**Authors:** Christine AF von Arnim, Stephanie Dismar, Cornelia S Ott-Renzer, Nathalie Noeth, Albert C Ludolph, Hans K Biesalski

**Affiliations:** 1 Department of Neurology, Ulm University, Oberer Eselsberg 45, 89081 Ulm, Germany; 2 Department of Biological Chemistry and Nutrition, Hohenheim University, Garbenstr. 30, 70593 Stuttgart, Germany

**Keywords:** B vitamins, Intracellular antioxidative status, Mini nutritional assessment, Micronutrient supplement

## Abstract

**Background:**

Malnutrition is a widespread problem in elderly people and is associated with cognitive decline. However, interventional studies have produced ambiguous results. For this reason, we wanted to determine the effect of micronutrient supplementation on blood and tissue levels and on general nutritional status in persons with mild or moderate cognitive impairment.

**Methods:**

We performed a 2-month, open-label trial, administering a daily micronutrient supplement to 42 memory clinic patients with mild cognitive deficits. Blood levels of antioxidants, zinc, and B vitamins were determined before and after supplementation. In addition, we assessed metabolic markers for B vitamins and intracellular (buccal mucosa cell [BMC]) antioxidant levels. Nutritional status was assessed by using the Mini Nutritional Assessment (MNA).

**Results:**

Blood levels of B vitamins, folic acid, lutein, β-carotene, α-carotene, and α-tocopherol increased significantly. Decreases in homocysteine levels and the thiamine pyrophosphate effect and an increase in holotranscobalamin were observed. We found no increase in intracellular antioxidant levels of BMC. The MNA score in subjects at risk for malnutrition increased significantly, mainly owing to better perception of nutritional and overall health status.

**Conclusions:**

Micronutrient supplementation improved serum micronutrient status, with improved metabolic markers for B vitamins but not for intracellular antioxidant status, and was associated with improved self-perception of general health status. Our data underline the necessity of determining micronutrient status and support the use of additional assessments for general health and quality of life in nutritional supplementation trials.

## Background

The aging population is increasing, and the risk of malnutrition increases with age [1]. As such, nutrition and nutritional supplementation for this population are becoming increasingly important. Older adults exhibit not only macronutrient deficiency but also micronutrient deficiency because demands for vitamins, minerals, and trace elements remain the same or are only slightly reduced in older people [2]. Deficiencies in folic acid, vitamin D, calcium, and vitamin B12 are especially prevalent [3,4]. Malnutrition may have an impact on cognitive function and, among patients with Alzheimer's disease (AD), cognitive and functional capacities worsen more rapidly in those who are most severely undernourished [5]. Several micronutrients, in particular vitamins B6 and B12 and folic acid with their effect on homocysteine (Hcy) metabolism [6-8], as well as antioxidants [9,10], have been shown to be associated with cognitive decline. Nevertheless, whether supplementation with micronutrients is capable of reducing the risk of dementia remains controversial [11-14]. Only a few trials of micronutrient supplementation in those who are cognitively impaired have been reported [15-18].

Little data is available regarding the absorption of nutritional supplements and their measurable effects on blood and tissue levels in elderly populations with mild or moderate cognitive impairment. To describe vitamin status, researchers commonly use serum or plasma concentrations; however, the information provided by these measurements is limited because data about tissue vitamin status are unreliable. Vitamin concentrations in blood can vary significantly and can be influenced by fasting, eating, or exercising [19-21]. Determination of intracellular levels would thus be advantageous for obtaining accurate knowledge about vitamin status.

Here we tested whether 2 months of micronutrient supplementation with a freely accessible over-the-counter supplement recommended for older adults leads to improved blood and intracellular micronutrient status in persons with mild or moderate cognitive impairment. As a secondary hypothesis, we used the Mini Nutritional Assessment (MNA) to assess whether supplementation can also improve general health and nutrition. To our knowledge, this is the first interventional trial in which novel methods were used to measure tissue vitamin levels.

## Methods

### Study design

The study was designed as an open-label, exploratory trial with micronutrient supplementation for 2 months in cognitively impaired elderly persons.

### Study population and baseline demographics

Forty-two (26 male [62%], 16 female [38%]) independently living older patients on a home-prepared diet, aged 61–87 years (mean 70.6 years), were recruited from the Memory Clinic of the University Hospital (Ulm, Germany), where they had been referred because of memory concerns. All participants were able to understand the implications and procedures of the study and gave written informed consent. Approval for this study was obtained from the ethics committee of the University of Ulm. Inclusion criteria were age above 60 years and only mild or moderate cognitive impairment. The study population had a Mini-Mental State Examination (MMSE) average score of 24.17 ± 6.91 and a Geriatric Depression Scale (GDS) average score of 3.26 ± 2.72. Anthropometric indicators included a mean baseline body mass index (BMI) of 26.95 ± 3.45 and a mean body weight of 76.15 ± 13.79. Exclusion criteria were acute illness, severe cognitive impairment, and severe depression. Participants with additional vitamin supplementation prior to study inclusion were excluded from the analysis of the supplemented vitamin (retinol: two participants; vitamin E: six; β-carotene, lutein, and lycopene: two; vitamin B1: two; vitamin B6: three; vitamin B12: six; folic acid: five; zinc: five). Participants were instructed to take one capsule of a micronutrient supplement daily after breakfast for 2 months.

### Composition of the micronutrient capsule

The micronutrient supplement (EunovaMultivitalstoffeLangzeit 50; GlaxoSmithKline, Bühl, Germany) is recommended for people aged ≥ 50 years and contains vitamins, minerals, and trace elements (Additional file 1: Table S1).

### Procedures

Before and after supplementation, subjects underwent the same examination procedures: blood and buccal mucosa cells (BMC) were collected. Nutritional assessments, MMSE, and GDS tests were administered. The participants were encouraged to maintain their dietary habits during the study period. A compliance telephone call took place 4–6 weeks after beginning the supplementation. Five participants dropped out prior to follow-up, so that 37 subjects completed the entire study. At the second visit, blisters were collected from the micronutrient supplements and the number of tablets taken were counted: 29 participants took exactly 60 tablets, six took 59, and two took 54, showing high compliance overall without significant differences in intake. Therefore we assessed all 37 participants as one group.

### Outcomes

The primary outcome was intracellular micronutrient status in BMC as well as in blood. The secondary outcome was assessment of general health and nutrition using the MNA.

### Collection of BMC and analysis of intracellular antioxidant status

Collection of BMC was performed as described previously [22] and samples were stored at −80°C. Vitamin concentrations (vitamin E, lycopene, β-carotene) of the samples were determined by BioTeSys GmbH (Esslingen, Germany) using an accredited high-performance liquid chromatography (HPLC) method in accordance with DIN EN ISO/IEC 17025.

### Sampling of blood and analytical methods

In addition to micronutrient blood levels, indirect markers of vitamin B status were determined to assess the metabolic effects of these vitamins. Subjects were instructed not to take a capsule on the day of the second examination, but did not fast because of the clinical setting. In vitamin B12 deficiency, the serum concentrations of methylmalonic acid (MMA) and Hcy increase. Hcy is also an indicator of folic acid deficiency. Holo-transcobalamin (TC) is the biologically available form of vitamin B12 [23,24]. A decrease in erythrocyte transketolase activity (ETKA) [25] or an increase in ETKA following stimulation with thiamine pyrophosphate (TPP) *in vitro* (TPP effect) represent improved markers for thiamine deficiency [26,27].

The levels of vitamins B1, B6, and B12; folic acid; zinc; albumin; and Hcy were analyzed by the clinical laboratory of the University Hospital. Concentrations of transketolase, the TPP effect, holo-TC, and MMA were determined by Synlab (Ulm, Germany). For determination of carotenoids, α-tocopherol, and retinol by HPLC, we used the method proposed by Neal Craft [28]. For the measurement of lutein, we used 8 μmol/L tocol as an internal standard and performed the analysis with reversed-phase HPLC in duplicate. For quantification, we used a plasma pool that was calibrated with the international certified standards from the National Institute of Standards and Technology.

Ascorbic acid (AA) and dehydroascorbic acid (DHA) were analyzed by reversed-phase HPLC. First, the plasma was thinned with 10% metaphosphoric acid 1:1 and then centrifuged (5 min, 4°C, 13000 *g*). The supernatant fraction was separated into two parts. Tris(2-chlorethyl)phosphate was added to one part to reduce the DHA contained therein and the samples were centrifuged again. After this step, the concentration of AA and total ascorbic acid (TAA = AA + DHA) could be detected. For quantification, two additional vitamin C standards (100 μM and 50 μM) were used. All samples were analyzed in duplicate. Reference values shown are in accordance with those from the respective laboratories or from previous publications [29].

### Assessment of nutritional status

To assess nutritional status, we determined both weight and BMI and applied the MNA. The 18-item questionnaire was developed especially for older adults [30]. In addition, we checked for general nutrition using a food questionnaire (Hohenheimer Kurzfragebogen), which showed no change in eating habits (Additional file 1: Table S2), as well as by measurement of albumin (albumin [g/l] before: 40.00 +/− 2.83; after 40.60 +/− 2.76; not significant). The MNA distinguishes three groups: those who are well nourished (MNA score ≥23.5), those with a risk of malnutrition (MNA score 17–23.5), and those with malnutrition (MNA score <17). MNA was performed in 41 participants before the study began and in 36 participants at the end of the study. To compare these scores, we included only those subjects with available MNA before and after supplementation.

### Sample size and statistical analysis

This pilot study was undertaken to determine initial data for the primary outcome measure in order to perform a sample size calculation for a larger trial. Sample size was therefore approximated by parameters to be estimated and aimed to include >30 participants. The total sample included 42 subjects before and 37 subjects after supplementation. All data were analyzed with SPSS (version 15.0; SPSS Inc., Chicago, USA). Normal distribution was checked by using a histogram and a q-q-plot. When there was normal distribution, the paired-samples t-test was used to detect significant differences in the same group, and the Wilcoxon test was used when the values had a skewed distribution. Additionally, Spearman’s correlation coefficient was taken to identify associations among the variables. P values < 0.05 were considered statistically significant for all tests.

## Results

### Effect of vitamin supplementation on micronutrient concentrations in blood and intracellular tissue

The first patient was enrolled in October 2008 and the last patient finished the study in March 2009. After excluding subjects with prior additional vitamin supplement use, we included those with a measured value before and after supplementation for analysis. Table 1 summarizes micronutrient levels in blood before and after supplementation. Most nutrients were well within the reference ranges or above, suggesting no overt malnutrition. However, before supplementation, seven participants had α- and β-carotene concentrations below the reference values. For zinc levels, 1 of 12 women had a concentration below the reference, whereas 14 (77.8%) men had a zinc concentration below the reference value. No significant change in zinc, retinol, or vitamin C was found after 2 months. A significant increase in plasma concentration after supplementation was, however, detected for α-carotene; β-carotene; vitamin E; lutein; vitamins B1, B6, and B12; and folic acid. After supplementation, all α-carotene levels were in the normal range. Only three persons had β-carotene levels below the reference value after supplementation. Lower levels of lycopene concentration were seen after supplementation, with the number of participants having lycopene levels under the reference value doubling afterward.

**Table 1:** Micronutrient levels in serum/plasma before and after supplementation are shown

***Micronutrient***	***Before *[*Mean *± *SD*]**	***After *** **[*Mean *± *SD*]**	***Reference value***	**P-value**
Vitamin C in plasma [μmol/L] N=31	61.22 ± 20.32	63.33 ± 12.0	45-80	n.s.
Vitamin A in plasma [μmol/L] N=35	2.21 ± 0.52	2.27 ± 0.40	> 1.05	n.s.
Vitamin E in plasma [μmol/L] N=31	36.55 ± 8.35	40.30 ± 8.68	> 16.2	p<0.05
α-Carotene in plasma [μmol/L] N=37	0.13 ± 0.12	0.15 ± 0.09	0.05–0.1	p<0.05
β-Carotene in plasma [μmol/L] N=35	0.58 ± 0.35	0.69 ± 0.47	0.3-0.6	p<0.01
Lutein in plasma [μmol/L] N=35	0.35 ± 0.16	0.40 ± 0.15	0.10–0.3	p<0.01
Lycopene in plasma [μmol/L] N=35	0.53 ± 0.22	0.45 ± 0.23	0.5–1.0	p<0.05
Vitamin B_1_ in serum [mmol/L] N=33	155.52 ± 49.22	187.73 ± 32.89	66–200	p<0.001
Vitamin B_6_ in serum [mmol/L] N= 32	83.72 ± 39.84	185.91 ± 65.56	35–110	p<0.001
Vitamin B_12_ in plasma [pmol/L] N=30	349.37 ± 117.08	382.40 ± 125.73	141–489	p<0.05
Folic acid in plasma [nmol/L] N=30	21.06 ± 8.64	39.8 ± 5.87	7–39.7	p<0.001
Zinc in plasma [μmol/L] N=12♀ / N=18♂	11.34± 1.45♀ 11.36 ± 1.46♂	11.83 ± 1.54♀ 11.03± 1.49♂	9.0–22.0♀ 12.0–26.0♂	n.s.

*n.s*. = not significant.

♀ = female. ♂ = male.

Table 2 shows the micronutrient concentrations in BMC before (n = 42) and after (n = 37) supplementation. Samples with concentrations below the detection level (n = 11) and with missing values (n = 24) before supplementation were excluded. Therefore, only seven patients were included for vitamin C analysis. We did not find significant changes in micronutrient concentrations in BMC. Of note, however, is that the number of patients with concentrations of vitamin C above the detection limit increased. Before supplementation, 11 (26.2%) subjects had a vitamin C concentration below the detection level, whereas afterward all measured vitamin C levels (n = 28) were above the detection level. To assess the intracellular vitamin supply in B vitamin metabolism, we analyzed specific metabolites; for vitamin B12, TC and MMA were used. Hcy increases in intracellular vitamin B6, vitamin B12, and folic acid deficiency. Low ETKA and a high TPP effect are markers for thiamine deficiency. TC, which carries the biologically available vitamin B12, increased significantly (P < 0.01) after supplementation (Table 3). Only two subjects had an MMA concentration > 47 μg/L, indicating vitamin B12 deficiency prior to supplementation. No significant decrease in MMA levels after supplementation was detected. After supplementation, Hcy levels decreased significantly (P < 0.001). Before supplementation, plasma levels of Hcy were elevated in 15 subjects, whereas afterward only five subjects had elevated Hcy concentrations. Although there was no significant change in ETKA, the decrease in the TPP effect was highly significant after supplementation.

**Table 2:** **Micronutrients in cells of buccal mucosa before** (**B**) **and after** (**A**) **supplementation are shown**

***Micronutrient***	***Before *** **(*B*) [*Mean *± *SD*]**	***After *** **(*A*) [*Mean *± *SD*]**	***Reference value***	**p-value**
BMC-Vitamin C [pmol/μgDNA] N=7	9.01 ± 5.72	11.76 ± 4.68	3.9–11.1	n.s.
BMC-Vitamin E [pmol/μg DNA] N=30	24.77 ± 9.56	26.45 ± 15.69	9.5–20.3	n.s.
BMC-β-Carotin [pmol/μg DNA] N=24	0.45 ± 0.44	0.40 ± 0.44	0.1–0.5	n.s.
BMC-Lycopin [pmol/μg DNA] N=19	0.37 ± 0.16	0.33 ± 0.25	0.1–0.5	n.s.

*n.s*. = not significant, *BMC* = buccal mucosa cell.

Reference values are given as listed below. No significant difference after supplementation was seen.

**Table 3:** **MMA**, **Holo**-**TC**, **Hcy**, **Transketolase**, **and the TPP**-**effect in serum**/**plasma before and after supplementation**

***Micronutrient***	***Before *** **(*B*) [*Mean *± *SD*]**	***After *** **(*A*) [*Mean *** **± *SD*]**	***Reference value***	**p-value**
Methylmalonic acid [μg/L] N=32	27.11 ± 13.68	26.87 ± 14.56	<47	n.s.
Holo-Transcobalamin [pmol/L] N=32	53.59 ± 21.35	61.47 ± 23.40	19,1-119,3	p<0.01
Homocysteine [μmol/L] N=29	12.87 ± 4.24	9.65 ± 2.79	5-12	p<0.001
Transketolase [U/L] N=35	62.48 ± 16.85	58.89 ± 8.89	60-80	n.s.
TPP-Effect [%] N=35	12.61 ± 7.46	6.14 ± 3.92	<20	p<0.001

*n.s*. = not significant, *TPP* = thiamin pyrophosphate.

### Effect of vitamin supplementation on nutritional and health status

Nutritional data are shown in Table 4. None of the volunteers was undernourished. Before supplementation, 25 (61%) subjects were well nourished, and 23 (63.9%) were afterward. Sixteen (39%) subjects were at risk for malnutrition before supplementation and 13 (36.1%) afterward. The risk for malnutrition was mainly attributable to a poor subjective assessment of health status, selection of food, or multi-medication. Comparison of the MNA scores of all 36 volunteers revealed no significant change (B: 24.27 ± 2.38; A: 24.64 ± 2.28). However, when the MNA scores of the well-nourished subjects and those at risk for malnutrition were analyzed separately, the score of the subjects at risk for malnutrition increased significantly after supplementation (B: 21.93 ± 1.45; A: 22.75 ± 1.25; P < 0.05), whereas the scores of the well-nourished subjects showed no difference (B: 25.76 ± 1.46; A: 25.84 ± 1.95). The MMSE scores were unchanged subsequent to supplementation.

**Table 4:** Nutritional status before and after supplementation

*** Micronutrient***	***Before *** **(*B*) *n*=** ***41 *[*Mean *** **± *SD*]**	***After *** **(*A*) *n*=*36 *** **[*Mean *** **± *SD*]**	**p-value**
Weight [kg]	76.15 ± 13.79	76.41 ± 13.64	n.s.
BMI [kg/m^2^]	26.95 ± 3.45	27.06 ± 3.35	n.s.
MNA [points]			
Well-nourished	25.76 ± 1.46	25.84 ± 1.95	n.s.
Risk of malnutrition	21.93 ± 1.45	22.75 ± 1.25	P <0.05
Undernutrition	-	-	n.s.

*n.s*. = not significant, *BMI*=Body mass index, *MNA*=Mini nutritional assessment.

To analyze changes in health status by supplementation, the MNA asks how test persons consider their health status in comparison with others of the same age. After supplementation, none of the participants reported their health status as “not as good,” whereas prior to supplementation, four (11.1%) did so. Furthermore, those who considered their health status as “better” increased by 16.6% and those who considered it to be “as good” decreased by approximately 8.3%. The improvement of the subjectively assessed health status was also the main reason why the subjects achieved better MNA scores after supplementation.

## Discussion

The purpose of this study was to assess the effect of a typical micronutrient supplement on specific blood and tissue nutrient levels as the primary outcome and on general nutritional status (assessed by MNA) as the secondary outcome in cognitively impaired, independently living older adults in a routine setting.

We did not see overt malnutrition in our patient population; however, several epidemiological studies have shown that in cognitive impairment and AD, lower levels of micronutrients within the normal range are observed than among controls, suggesting that there is an additional need for specific micronutrients. For zinc, we observed a micronutrient deficiency at baseline only in men. Our data show, however, that a significant improvement in selected micronutrients can be achieved after taking a micronutrient supplement for 2 months.

### Effect of vitamin supplementation on micronutrient concentrations in blood and intracellular tissue

Our data are consistent with those of others in showing decreased zinc levels in men with mild cognitive impairment [31]. This could be explained by the fact that men have a higher daily zinc requirement (10 mg/day) than women (7 mg/day). Nonetheless, after supplementation, plasma zinc levels did not increase. One possible reason could be decreased zinc absorption efficiency in elderly persons [32]. Inasmuch as a single capsule contains only 3.75 mg of zinc (54% of the daily requirement for women and only 38% for men), this amount might not be sufficient. A second explanation might be that the study period (2 months) might have been too short for zinc regarding its turnover time. One study has shown that improving the zinc status of older adults (> 65 years) with low zinc levels by using a supplement (7 mg zinc) for 1 year resulted not only in a decreased incidence and duration of pneumonia, but also in a decreased number of new antibiotic prescriptions and decreased total days of antibiotic use [33]. In addition, normal baseline serum zinc concentrations were associated with a reduction in all causes of mortality.

In our study, the micronutrient status of vitamin E, β-carotene, and lycopene in BMC was within normal limits in most subjects before and after supplementation. With regard to vitamin C, only seven BMC concentrations were above detection levels despite normal vitamin C values in plasma at baseline. After intervention, vitamin C was above detection levels in all patients, indicating that 150 mg (250% of recommended dietary allowance [RDA]) is sufficient. The plasma and BMC concentrations of β-carotene and lycopene were significantly correlated before and after supplementation. Similarly, a study by Peng et al. also found a correlation between these micronutrients in plasma and BMC [34]. On the other hand, Pateau et al. found no correlation between the lycopene concentration in plasma and BMC [35]. Because the recommended daily amount of β-carotene is 2–4 mg and no recommendation for lycopene exists, and because the supplement contained only 0.5 mg of carotenoids (<25% RDA), our data may support the need for increased levels of carotenoids and lycopenes in supplements. Plasma and BMC concentrations of vitamin E were significantly correlated before supplementation, similar to the findings reported in Peng et al. [34]. Following supplementation, this correlation was lacking, indicating that BMC were completely saturated after supplementation, whereas plasma concentration is not saturable. This may suggest that the level of vitamin E in the supplement with 200% RDA is more than sufficient.

Although the subjects had normal B vitamin levels in blood, 15 (51.7%) displayed increased Hcy levels before supplementation, indicating that blood levels of vitamin B6, vitamin B12, and folic acid inadequately reflect intracellular supply. We observed an improvement in B vitamin status and a subsequent decrease in plasma Hcy. Hcy is not only a risk factor for vascular disease, but also for cognitive disorders [7]. Quadri et al. found an association between dementia and hyperhomocysteinemia [36]. Many studies found that elderly people in particular have a deficiency in their supplies of vitamin B12 and folic acid [37]. In our study, most blood levels for vitamins B1, B6, and B12 and for folic acid were normal before supplementation. After supplementation, these levels increased significantly. This is in line with the amount of vitamins contained in the supplement itself: B1 (200% RDA), B6 (>300% RDA), B12 (84% RDA), and folic acid (125% RDA). Whereas there was no significant change in the concentration of MMA, holo-TC increased significantly (P < 0.01), indicating that the intracellular status could still be optimized. Thus, an increase in the amount of vitamin B12 in the supplement may help to achieve better efficacy. However, analysis of the required amount of supplementation requires further study with several levels of supplementation.

Despite there being normal vitamin B1 concentrations in the blood, the low ETKA in 18 (51.4%) subjects indicated B1 deficiency in tissue. This finding was supported by an elevated TPP effect of more than 20% in some of our patients. Decreased ETKA might not be the best indicator in the elderly population owing to apoenzyme variations. In a study comparing ETKA and the TPP effect in healthy elderly subjects (70–82 years of age) with those in young subjects (19–37 years of age), researchers found that the older adults had a lower ETKA but a similar TPP effect and erythrocyte thiamine levels [38]. Although the vitamin B1 concentration in blood increased significantly (P < 0.001) after supplementation in our study, ETKA did not increase. The TPP effect, however, decreased significantly, indicating improved intracellular vitamin B1 status and confirming the superiority of measuring the TPP effect.

Taken together, our results showed decreased Hcy levels, a decreased TPP effect, and improved antioxidant status after micronutrient supplementation. This might be helpful for improving cognitive function. The FACIT study, which showed beneficial effects on global cognitive function in men and women (aged 50–70 years), achieved decreased Hcy levels following 3-year supplementation with folic acid [13]. A multivitamin, multimineral-enriched drink administered over a 6-month period decreased Hcy levels, increased B12 levels, and improved neuropsychological performance in frail elderly persons (N = 101; age > 65 years; BMI <25) [39]. Supplementation with a multinutrient drink for 12 weeks improved memory (delayed verbal recall) in patients with mild AD [15]. In our study, the period of supplementation was probably too short to have a positive effect on cognition as measured by MMSE.

### Effect of vitamin supplementation on nutritional and health status

Micronutrient supplementation was associated with a significant increase in the MNA score in persons at risk for malnutrition. This improvement was mainly attributable to improved self-perception of general health status after micronutrient supplementation. The five dropouts in the MNA analysis may also have affected this result, especially considering the open-label character of the study. The MNA is a well-established assessment tool for the identification of patients at risk for malnutrition regardless of cognitive condition [40] and has also been used in interventional studies for follow-up evaluation [41,42]. The MNA questions that showed significant intercorrelations in the study by Soini et al. [42] formed logical patterns between anthropometrics, decline in food intake with weight loss, self-perceived nutritional status, the ability to eat, and the use of fruit and vegetables. However, the results of the MNA should not be overemphasized, because the aim of this supplementation was not the improvement of energy and protein deficiency. Other studies have shown that subjective assessment of health does have predictive value [43]. Christensson et al. [44] concluded that “self-experienced health status” has the greatest predictive value in MNA classification. One reason they discussed was the possibility of a reduced incidence of infections. Barringer et al. [45] found a significantly reduced incidence of infections in patients with diabetes (age > 65) after 1 year of multivitamin and mineral supplementation. In Christensson et al. [44], quality of life was assessed as a secondary endpoint by the Medical Outcomes Study 12-Item Short Form, which showed no difference before and after supplementation. A significant risk reduction for infections was shown after 18 months of multivitamin supplementation in a subgroup of patients without dementia [46]. We did not assess infection rate in the present study. We propose that in future trials, an assessment for infections and for comprehensive quality of life should be incorporated.

### Strengths and limitations

The main strength of this study is its comprehensive approach for the determination of blood and intracellular nutrient status, as well as assessment of metabolic markers before and after supplementation, in addition to general nutritional assessment. To our knowledge, the comprehensive and innovative methods used herein for micronutrient analysis are novel.

One limitation is that we did not analyze all of the micronutrients contained in this trial supplement. In addition, the generalizability of our results is limited because of the sample size. Moreover, without parallel controls, the primary outcome could not be separated from a time effect and the secondary outcome could not be separated from a placebo effect. A major limitation regarding self-assessed health status is the open-label character of this study, which could have biased the subjective assessment of the participants.

## Conclusions

First, our data support the idea that micronutrient supplements need to be carefully analyzed and adjusted for specific groups. Our data indicate that vitamin B1, vitamin B12, lycopene, and carotenoids should be given at higher doses than used here to optimize their supply. Second, placebo-controlled studies with longer periods of micronutrient supplementation in elderly persons at risk for malnutrition are required to further investigate the effects of micronutrient supplements on nutritional status and on quality of life.

## Competing interests

C.A.F. von Arnim has received honoraria from Pfrimmer-nutricia, Pfizer, Novartis, and Eisai. This study was supported by Day med concept, Berlin, Germany.

## Authors’ contributions

SD, CSO-R, and NN examined the participants; participated in the design of the study and the analysis and interpretation of the data; and drafted the manuscript. SD and CSO-R. participated in the biochemical analysis. HKB, ACL, and CAFvA. conceived the study; participated in its design; analyzed and interpreted the data; and coordinated and helped draft the manuscript. All authors read and approved the final manuscript.

## Supplementary Material

Additional file 1: Table S1 Composition of a single capsule of EUNOVA 50+. * Recommended daily allowance (RDA) according to the EU Nutritional Value Labeling Directive 90/496/EEC. ** No EU recommendation existent. **Table S2.** Changes in intake of vegetables/vitamins, protein, and carbohydrates. Numbers of patients with specific frequency of indicated food intake before and after study. Click here for file
