# Supplementary material for: Micronutrients supplementation and nutritional status in cognitively impaired elderly persons: a two-month open label pilot study
Source: Nutr J. 2013 Nov 15;12:148. doi: 10.1186/1475-2891-12-148 (PMC3834880; doi:10.1186/1475-2891-12-148)
Supplement: Additional file 1: Table S1 — Composition of a single capsule of EUNOVA 50+. * Recommended daily allowance (RDA) according to the EU Nutritional Value Labeling Directive 90/496/EEC. ** No EU recommendation existent. Table S2. Changes in intake of vegetables/vitamins, protein, and carbohydrates. Numbers of patients with specific frequency of indicated food intake before and after study. [file 1475-2891-12-148-S1.doc]

Supplemental table 1 **Composition of a single capsule of EUNOVA 50+.** * Recommended daily allowance (RDA) according to the EU Nutritional Value Labeling Directive 90/496/EEC. ** No EU recommendation existent.

| **Component** | **Amount per capsule** | **RDA *** |
| --- | --- | --- |
| Vitamin A | 600 μg RE | 75 % |
| Carotenoides (β-Carotene, α-Carotene, γ-Carotene, zeaxanthin) | 0.6mg | ** |
| Lutein | 0.75 mg | ** |
| Lycopene | 0.5 mg | ** |
| Vitamin C | 150 mg | 250 % |
| Vitamin E | 22.5 mg | 225 % |
| Vitamin B1 | 3.5 mg | 250 % |
| Vitamin B2 | 4.0 mg | 250 % |
| Vitamin B6 | 5.0 mg | 250 % |
| Vitamin B12 | 2.5 μg | 250 % |
| Biotin | 375 μg | 250 % |
| Folic acid | 500 μg | 250 % |
| Niacin | 45 mg | 250 % |
| Pantothenic acid | 12 mg | 200 % |
| Vitamin D3 | 3.75 μg | 75 % |
| Vitamin K | 18.75 μg | ** |
| Chloride | 6.75 | ** |
| Potassium | 7.5 mg | ** |
| Manganese | 0.68 mg | ** |
| Chrome | 18.9 μg | ** |
| Molybdenum | 15 μg | ** |
| Selenium | 7.5 µg | ** |
| Iodine | 75 μg | 50 % |
| Zinc | 3.75 mg | 25 % |

Supplemental table 2 **Changes in intake of vegetables/vitamins, protein, and carbohydrates.** Numbers of patients with specific frequency of indicated food intake before and after study.

| **food category** | **several times a day** | | **once daily** | | **4-6/week** | | **1-3/week** | | **rarely** | | **never** | |
| --- | --- | --- | --- | --- | --- | --- | --- | --- | --- | --- | --- | --- |
|  | before | after | before | after | before | after | before | after | before | after | before | after |
| **vegetable/vitamins** |  |  |  |  |  |  |  |  |  |  |  |  |
| fruit | 17 | 18 | 12 | 14 | 1 | 1 | 4 | 1 | 3 | 3 | 0 | 0 |
| vegetables | 8 | 4 | 23 | 24 | 3 | 2 | 3 | 6 | 0 | 0 | 0 | 0 |
| **protein** |  |  |  |  |  |  |  |  |  |  |  |  |
| meat | 0 | 0 | 0 | 0 | 9 | 9 | 24 | 25 | 4 | 3 | 0 | 0 |
| fish | 0 | 0 | 0 | 0 | 0 | 0 | 18 | 17 | 18 | 19 | 1 | 1 |
| poultry | 0 | 0 | 0 | 0 | 0 | 0 | 9 | 6 | 28 | 31 | 0 | 0 |
| sausage | 7 | 5 | 20 | 20 | 4 | 6 | 4 | 4 | 2 | 2 | 0 | 0 |
| cheese | 1 | 1 | 19 | 19 | 4 | 6 | 11 | 10 | 2 | 1 | 0 | 0 |
| dairy products | 5 | 5 | 13 | 12 | 1 | 1 | 13 | 13 | 5 | 6 | 0 | 0 |
| eggs | 0 | 0 | 0 | 1 | 0 | 0 | 19 | 19 | 15 | 17 | 3 | 0 |
| **carbohydrates** |  |  |  |  |  |  |  |  |  |  |  |  |
| grain/flour | 33 | 33 | 4 | 4 | 0 | 0 | 0 | 0 | 0 | 0 | 0 | 0 |
| beans/legumes | 0 | 0 | 0 | 0 | 0 | 0 | 11 | 11 | 24 | 25 | 2 | 1 |
